# Supplementary material for: The KLF7/PFKL/ACADL axis modulates cardiac metabolic remodelling during cardiac hypertrophy in male mice
Source: Nat Commun. 2023 Feb 21;14:959. doi: 10.1038/s41467-023-36712-9 (PMC9944323; doi:10.1038/s41467-023-36712-9)
Supplement: Supplementary file 1 — Supplementary Information [file 41467_2023_36712_MOESM1_ESM.pdf]

The KLF7/PFKL/ACADL axis modulates cardiac metabolic remodelling during cardiac hypertrophy in male mice

Cao Wang[1], Shupei Qiao[2], Yufang Zhao[3], Hui Tian[1], Wei Yan[4], Xiaolu Hou[5], Ruiqi Wang[1], Bosong Zhang[1], Chaofan Yang[1], Fuxing Zhu[1], Yanwen Jiao[1], Jiaming Jin[1], Yue Chen[1], Weiming Tian[1],*

[1]School of Life Science and Technology, Harbin Institute of Technology, Harbin 150080, China

[2]NHC and CAMS Key Laboratory of Molecular Probe and Targeted Theranostics, Harbin Medical University, Harbin, 150081，China

[3]Space Environment Simulation Research Infrastructure, Harbin Institute of Technology, Harbin 150080, China

[4]Department of Cardiology, The First Affiliated Hospital of Harbin Medical University, Harbin, 150081，China

[5]Department of Cardiology, The Fourth Affiliated Hospital of Harbin Medical University, Harbin, 150081，China

These authors contributed equally: Cao Wang, Shupei Qiao

*Correspondence: tianweiming@hit.edu.cn

Tel.:+86-18545558869

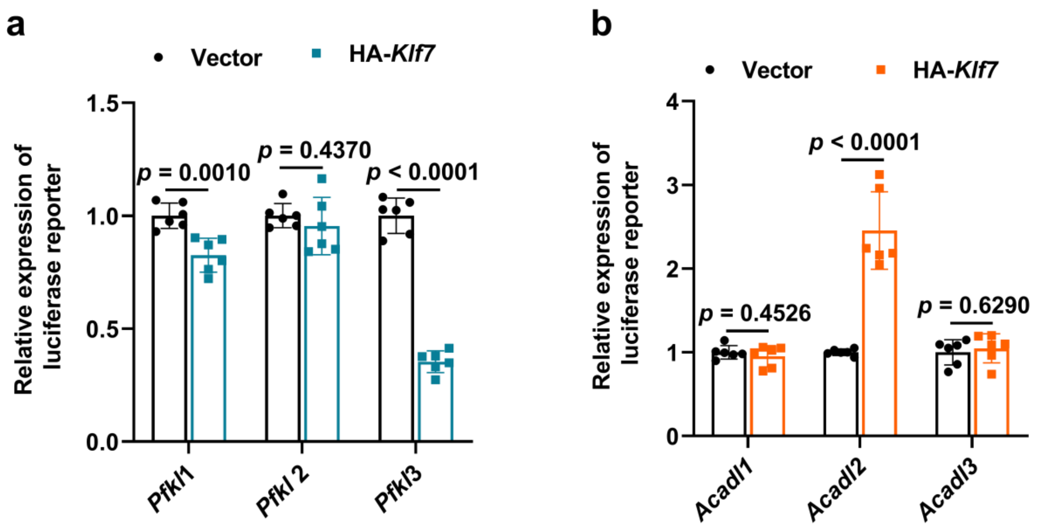

Supplementary Fig. 1. *Klf7* targets the promotor regions of *Pfkl* and *Acadl*. a Analysis of a specific 250-bp sequence in the *Pfkl* promoter region that can negatively target binding to the *Klf7* gene (n = 6 biologically independent experiments). **b** Analysis of a specific 180-bp sequence in the *Acadl* promoter region that can positively target binding to the *Klf7* gene (n = 6 biologically independent experiments). Two-tailed unpaired Student's t-test in **a** and **b**. Data are depicted as the mean values ± SEM. Source data are provided as a Source Data file.

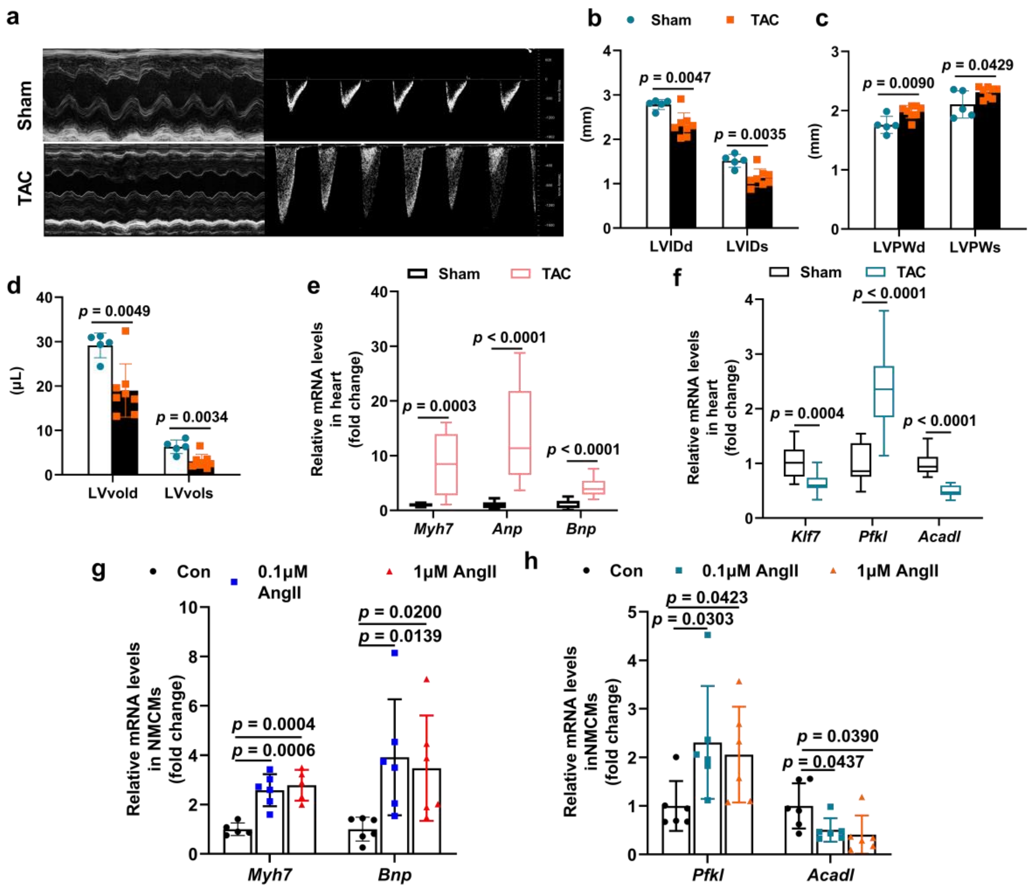

Supplementary Fig. 2. Scheme showing construction of the cardiac hypertrophy model in vivo and in vitro. **a** Representative examples of M-mode and Doppler echocardiograms from adult WT mice under pressure overload at 4 weeks after TAC. **b-d** Compared with the corresponding parameters in TAC mice and sham mice, the end-diastolic left ventricle inner diameter was decreased, the end-diastolic posterior wall thickness was increased, and the end-diastolic LV volume was decreased (n = 5 mice/group). **e** The expression of cardiac fetal genes was upregulated in the hearts of TAC mice (n = 15 biologically independent experiments). **f** Cardiac hypertrophic growth induced by TAC downregulated the expression of *Klf7*, upregulated *Pfkl* expression and downregulated *Acadl* expression at the mRNA level, as assessed by qRT-PCR (n = 15 biologically independent experiments). **g** The mRNA levels of *Bnp* and *Myh7* were upregulated in NMCMs 48 hours after AngII treatment (n = 6

biologically independent experiments). **h** The mRNA levels of *Pfkl* were upregulated, and those of *Acadl* were downregulated in NMCMs 48 hours after AngII treatment (n = 6 biologically independent experiments). **e** and **f**, The boxplot represents the median shown as a line in the center of the box, the boundaries are the first and third quartile, and whiskers represent the minimum and maximum values in the data. Two-tailed unpaired Student's t-test in **b**-**f**. One-way ANOVA with Tukey's multiple comparison test in **g** and **h**. Data are depicted as the mean values ± SEM. TAC, transverse aortic constriction. Source data are provided as a Source Data file.

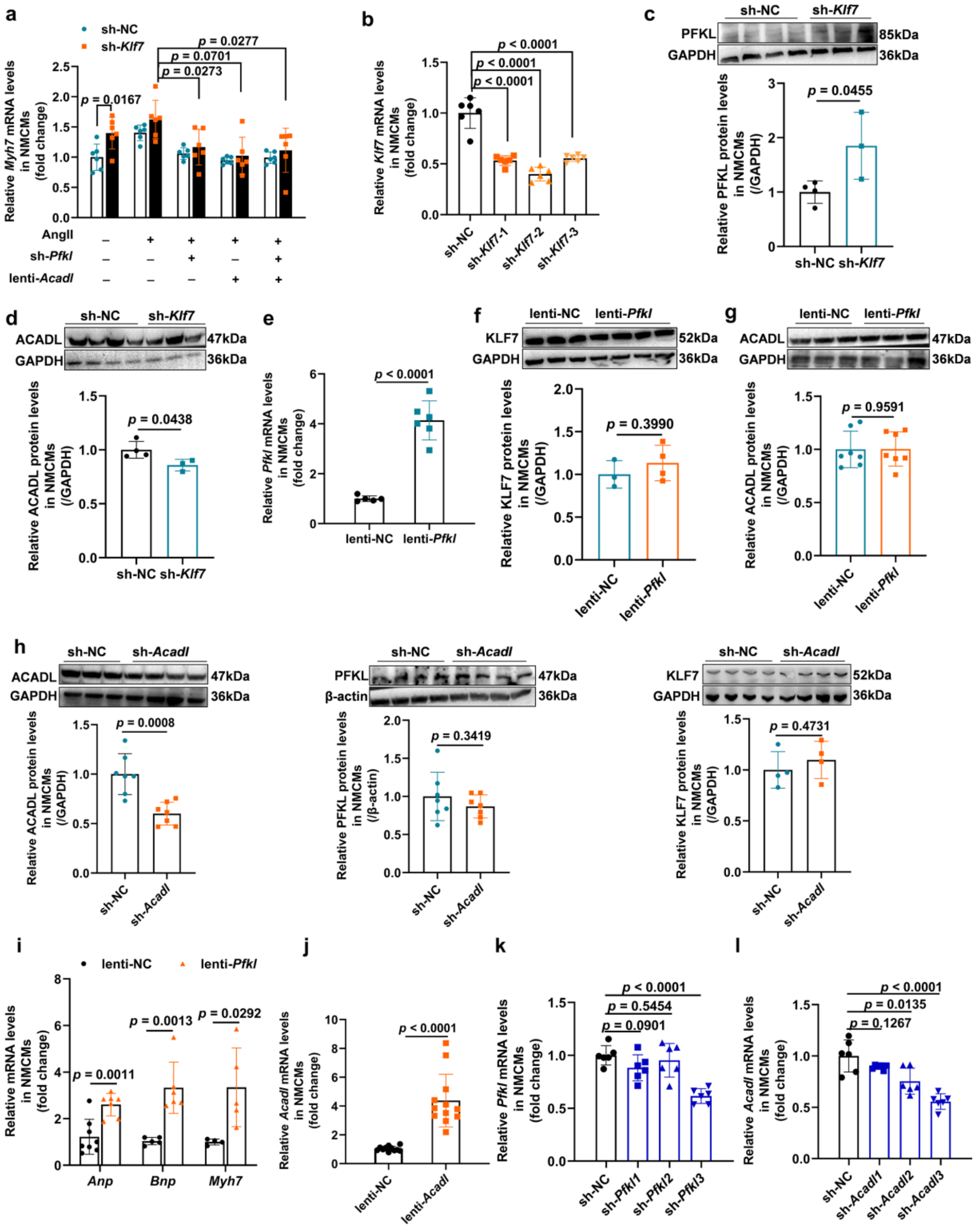

Supplementary Fig. 3. Detection of the infection efficiency of lentiviruses used for *Pfkl*, *Acadl* and *Klf7* expression. **a** Quantification of *Myh7* expression in NMCMs transduced with sh-*Klf7*, sh-*Pfkl*, lenti-*Acadl* or a combination of sh-*Pfkl* and lenti-*Acadl* as indicated. *Pfkl* knockdown, *Acadl* overexpression and both at the same time were sufficient to attenuate hypertrophic growth induced by knockdown *Klf7* (n = 6 biologically independent experiments). **b** The *Klf7* mRNA knockdown efficiency

was 60% in NMCMs transfected with sh-*Klf7*-2 (n = 6 biologically independent experiments). **c**, **d** The PFKL and ACADL protein expression was increased and decreased in cardiomyocytes transduced with sh-*Klf7* or sh-NC as indicated, respectively (sh-NC, n = 4 biologically independent experiments, sh-*Klf7*, n = 3 biologically independent experiments). **e** The *Pfkl* mRNA overexpression efficiency was 4 times in NMCMs transfected with lentivirus (n = 6 biologically independent experiments). **f**, **g** Analysis of KLF7 and ACADL protein in NMCMs treated with lenti-*Pfkl* and lenti-NC (**f**, lenti-NC, n = 3 biologically independent experiments, lenti-*Pfkl*, n = 4 biologically independent experiments; **g**, n = 7 biologically independent experiments). **h** Protein expression analysis of ACADL, KLF7 and PFKL expression in sh-*Acadl* and sh-NC (ACADL, n = 7 biologically independent experiments; PFKL, n = 7 biologically independent experiments; KLF7, n = 4 biologically independent experiments). **i** qRT-PCR showed that the mRNA levels of *Anp*, *Bnp* and *Myh7* were upregulated in NMCMs transfected with lenti-*Pfkl* and lenti-NC (n = 6 biologically independent experiments). **j** *Acadl* mRNA levels were 6 times higher in NMCMs transfected with lenti-*Acadl* (n = 10 biologically independent experiments). **k** Assessment of knockdown efficiency revealed that *Pfkl* mRNA levels were 35% of the control value in NMCMs transfected with sh-*Pfkl*-3 (n = 6 biologically independent experiments). **l** *Acadl* mRNA levels were 40% of the control value in NMCMs transfected with sh-*Acadl*-3 (n = 6 biologically independent experiments). Two-tailed unpaired Student's t-test in **a**, **b**, **k** and **l**. One-way ANOVA with Tukey's multiple comparison test in **c**-**j**. Data are depicted as the mean values ± SEM. sh, short hairpin; lenti, lentivirus overexpression. Source data are provided as a Source Data file.

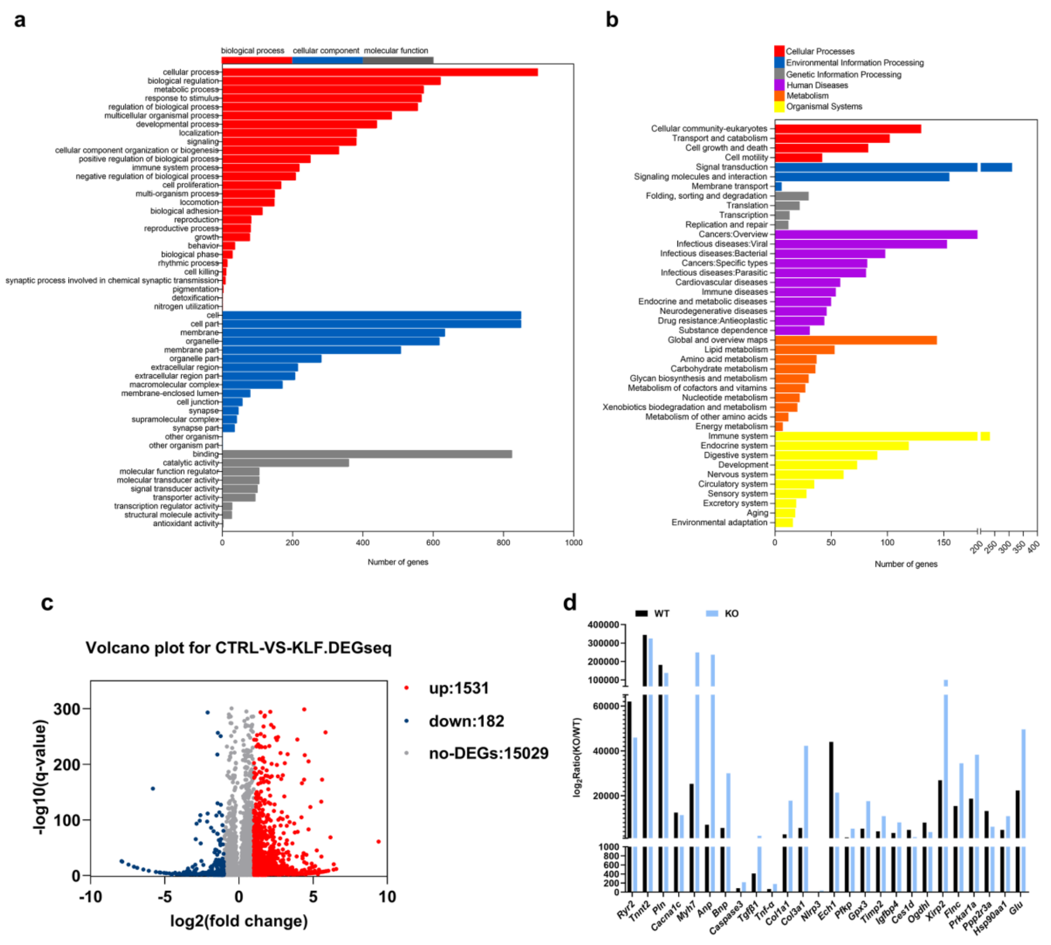

Supplementary Fig. 4. Cardiac-specific knockout *Klf7* disturbed the expression of genes involved in cardiac glucose and fatty acid metabolism. a GO annotation of the DEGs from KO and WT mouse hearts. The X axis indicates the GO term. The Y axis indicates the number of up/downregulated genes (n = 3 mice/group). **b** Classification of KEGG pathway enrichment. The X axis indicates the number of DEGs. The Y axis indicates the functional KEGG pathway. Seven branches of KEGG pathways were revealed: Cellular Processes, Environmental Information Processing, Genetic Information Processing, Human Disease (for animals only), Metabolism, Organismal Systems and Drug Development (n = 3 mice/group). **c** MA plot following DEGs distribution analysis of the cardiac tissues of KO and WT mice. The *x* axis indicates value A (log2-transformed mean expression level). The *y* axis indicates value M (log2-transformed fold change). Differentially expressed genes were

identified with the threshold of $|\log2(\text{fold change})| > 1$ and false discovery rate (FDR) <0.05. Red dots represent upregulated DEGs. Blue dots represent downregulated DEGs. Grey points represent non-DEGs (n = 3 mice/group). **d** The top most significant DEGs in the KO and WT mice, including genes related to cardiac hypertrophy, glucose and lipid metabolism, cardiac contractile function and the extracellular matrix (n = 3 mice/group). DEGs, differentially expressed genes. KO, knockout. Two-tailed unpaired Student's t-test in **c**. Data are depicted as the mean values ± SEM. Source data are provided as a Source Data file.

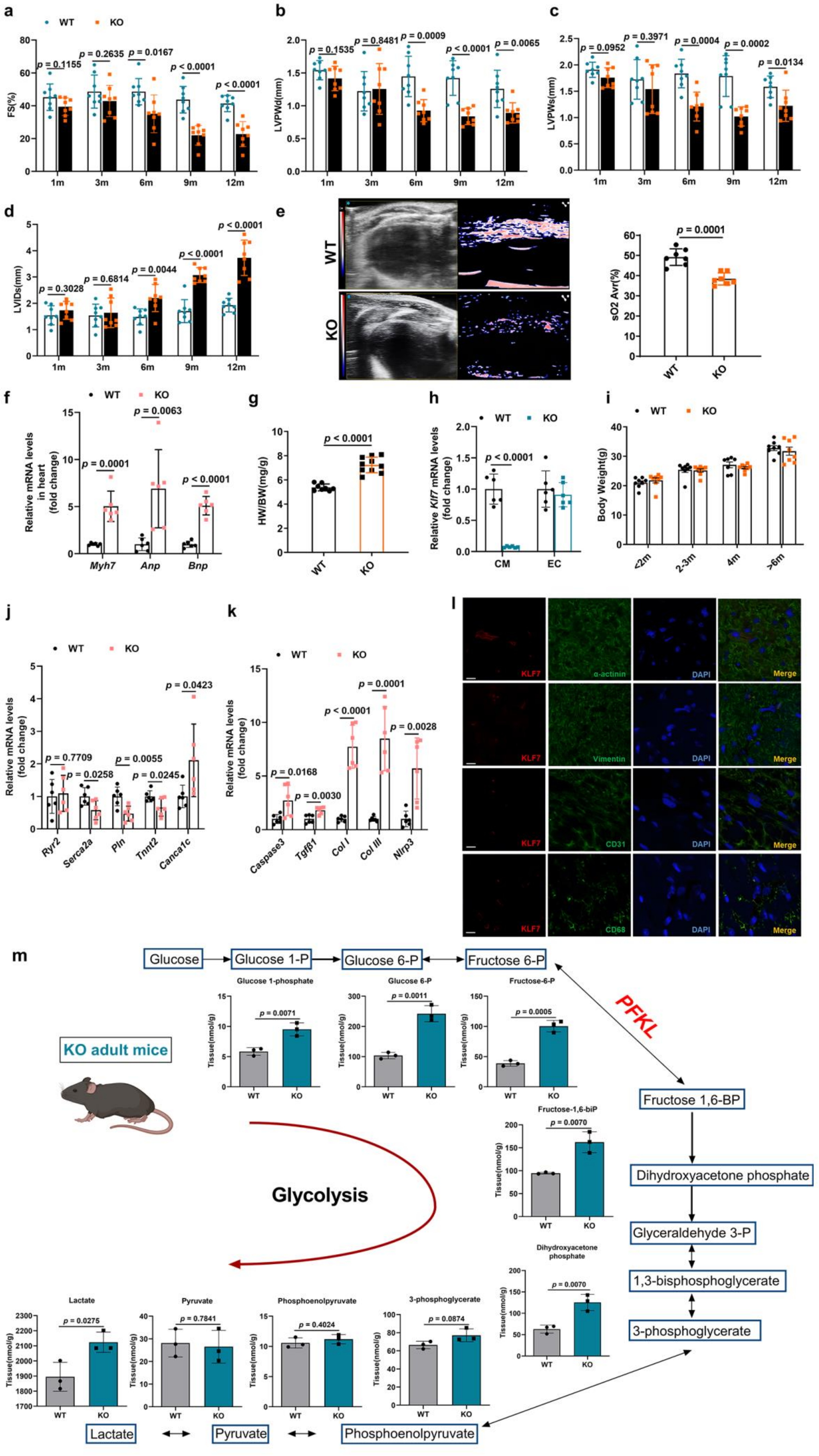
a
WT
KO
FS(%)
p = 0.1155
p = 0.2635
p = 0.0167
p < 0.0001
p < 0.0001
1m
3m
6m
9m
12m
b
LVPWd(mm)
p = 0.1535
p = 0.8481
p = 0.0009
p < 0.0001
p = 0.0065
c
LVPWs(mm)
p = 0.0952
p = 0.3971
p = 0.0004
p = 0.0002
p = 0.0134
d
LVIDs(mm)
p = 0.3028
p = 0.6814
p = 0.0044
p < 0.0001
p < 0.0001
e
WT
KO
sO2 Avr(%)
p = 0.0001
f
Relative mRNA levels in heart (fold change)
p = 0.0001
p = 0.0063
p < 0.0001
Myh7
Anp
Bnp
g
HW/BW(mg/g)
p < 0.0001
h
Relative Klf7 mRNA levels (fold change)
p < 0.0001
CM
EC
i
Body Weight(g)
<2m
2-3m
4m
>6m
j
Relative mRNA levels (fold change)
p = 0.7709
p = 0.0258
p = 0.0055
p = 0.0245
p = 0.0423
Ryr2
Serca2a
Pln
Tnnt2
Canca1c
k
Relative mRNA levels (fold change)
p = 0.0168
p = 0.0030
p < 0.0001
p = 0.0001
p = 0.0028
Caspase3
Tgfβ1
Col I
Col III
Nlrp3
l
KLF7
α-actinin
Vimentin
CD31
CD68
DAPI
Merge
m
Glucose
Glucose 1-P
Glucose 6-P
Fructose 6-P
Glucose 1-phosphate
p = 0.0071
Glucose 6-P
p = 0.0011
Fructose-6-P
p = 0.0005
Tissue(nmol/g)
PFKL
KO adult mice
Fructose 1,6-BP
Fructose-1,6-biP
p = 0.0070
Dihydroxyacetone phosphate
p = 0.0070
Glyceraldehyde 3-P
1,3-bisphosphoglycerate
3-phosphoglycerate
Glycolysis
Lactate
p = 0.0275
Pyruvate
p = 0.7841
Phosphoenolpyruvate
p = 0.4024
3-phosphoglycerate
p = 0.0874
Lactate
Pyruvate
Phosphoenolpyruvate

Supplementary Fig. 5. Cardiac-specific *Klf7* deficiency is critical for cardiac dysfunction and even HF with age. When KO mice and WT mice of different ages were compared, **a** fraction shortening (FS) was decreased (n = 8 mice/group), **d** end-systolic left ventricle inner diameter (LVID;s) was increased (n = 8 mice/group), and **b**, **c** end-diastolic and systolic posterior wall thickness (LVPW;d) was decreased in the KO mice (n = 8 mice/group). **e** Representative oxy-haemo photoacoustic images used to analyze the decrease in blood oxygen saturation (%sO2) of the ventricular wall of 9-month-old mice. The heat map indicates blood oxygen saturation levels ranging from 0% (dark blue) to 100% (dark red) (n = 7 mice/group). **f** Relative quantification showed increases in fetal gene expression (*Anp*, *Bnp*, *Myh7*) (n = 6 biologically independent experiments). **g** The HW/BW was increased in 9-month-old KO mice (n = 10 biologically independent experiments). **h** *Klf7* knockout efficiency in KO mice was about 93% in flow-sorted cardiomyocytes was assessed by qRT-PCR (n = 6 biologically independent experiments). **i** There was no differences in body weight between KO mice and WT mice at the same age (n = 8 mice/group). **j** The expression of cardiac function-related genes (*Ryr2*, *Serca2a*, *Pln*, *Tnnt2*, *Cacna1c*) was disordered in the hearts of KO and WT mice (n = 6 biologically independent experiments). **k** The expression of fibrosis marker genes (*Caspase3*, *Tgfβ1*, *Col I*, *Col III*, *Nlrp3*) was upregulated in the hearts of *Klf7*^{KO} and WT mice (n = 6 biologically independent experiments). **l** Hearts of WT mice were immunofluorescent stained to determine of *Klf7* (red) in α-actin (cardiomyocytes), Vimentin (cardiac fibroblasts), CD31 (endothelial cells) and CD68 (macrophages)-positive cells. (n = 3 mice/group, 6 sections/mouse); Scale bar = 10 μm. **m** Pathway analysis of data obtained from mass spectrometry-based metabolomics showed that the knockout of *Klf7* significantly increased glycolytic intermediates (n = 3 biologically independent

samples). Two-tailed unpaired Student's t-test in **e**-**h**, **j**, **k** and **m**. Two-way ANOVA with Tukey's multiple comparison test in **a**-**d**, and **i**. Data are depicted as the mean values ± SEM. Source data are provided as a Source Data file.

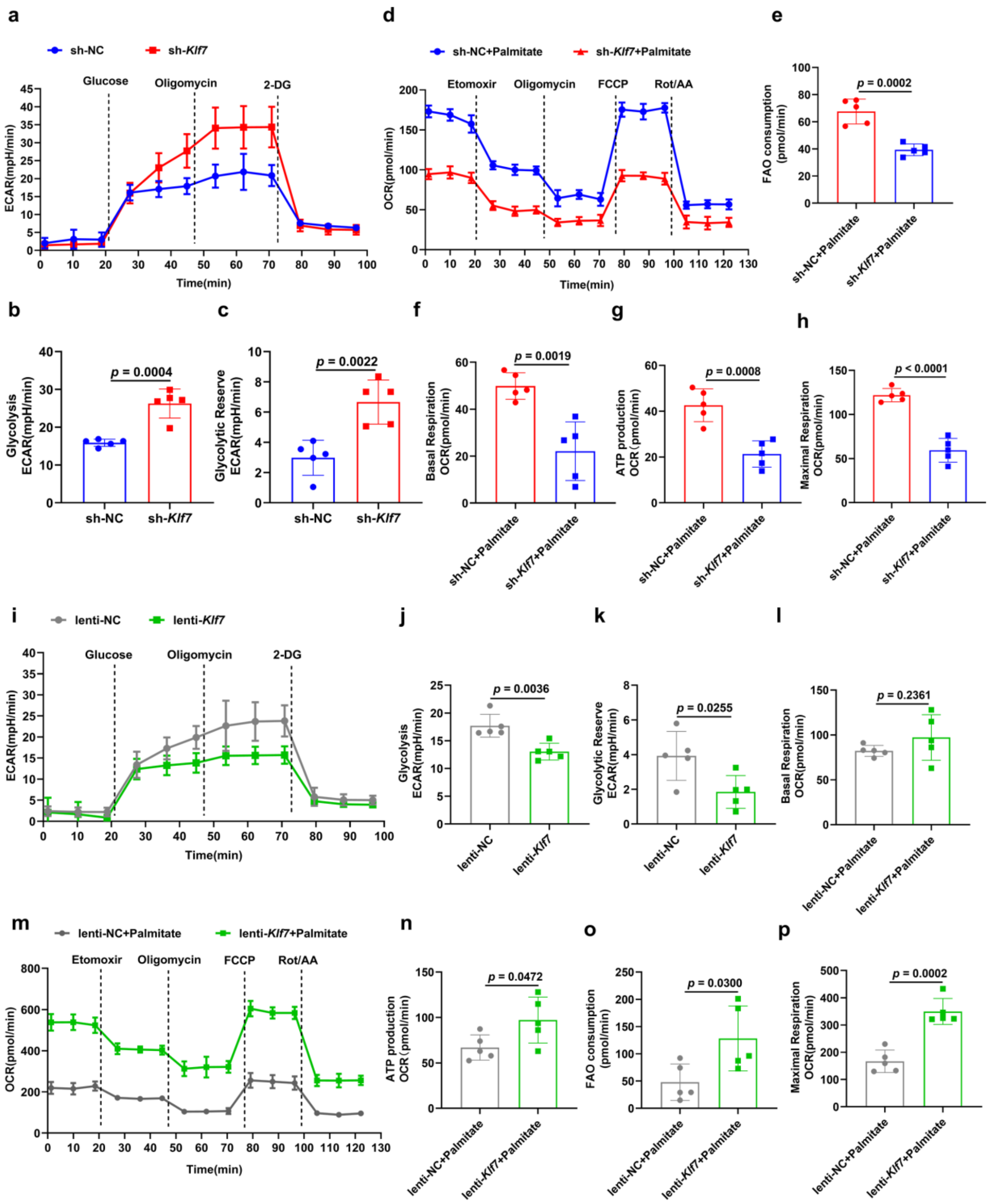

Supplementary Fig. 6. *Klf7* regulates both glycolysis and FAO capacity in cardiomyocytes. **a-c** Knockdown of *Klf7* in NMCMs have enhanced glycolysis and glycolytic reserve (n = 5 biologically independent experiments) **d** OCR from a representative experiment where each point of 5 replicates in knocking down KLF7 with lentivirus (sh-*Klf7*) and controls (sh-NC) (n = 5 biologically independent experiments). **e** FAO consumption. **f** Basal respiration. **g** ATP production. **h** Maximal respiration in NMCMs treated with sh-*Klf7* and sh-NC (n = 5 biologically independent experiments). **m** OCR in overexpression of KLF7 with lentivirus (lenti-

Klf7) and controls (lenti-NC) (n = 5 biologically independent experiments). **i-k** Overexpression of *Klf7* in NMCMs have reduced glycolysis and glycolytic reserve (n = 5 biologically independent experiments). **m** OCR from a representative experiment where each point of 5 replicates in overexpression *Klf7* with lentivirus (lenti-*Klf7*) and controls (lenti-NC) (n = 5 biologically independent experiments). **o** FAO consumption. **l** Basal respiration. **n** ATP production. **p** Maximal respiration in NMCMs treated with lenti-*Klf7* increased compared to lenti-NC (n = 5 biologically independent experiments). Two-tailed unpaired Student's t-test in **b**, **c**, **e**-**h**, **j**-**l** and **n**-**p**. Data are depicted as the mean values ± SEM. sh, short hairpin; lenti, lentivirus overexpression; ECAR, extracellular acidification rate; OCR, oxygen consumption rate. Source data are provided as a Source Data file.

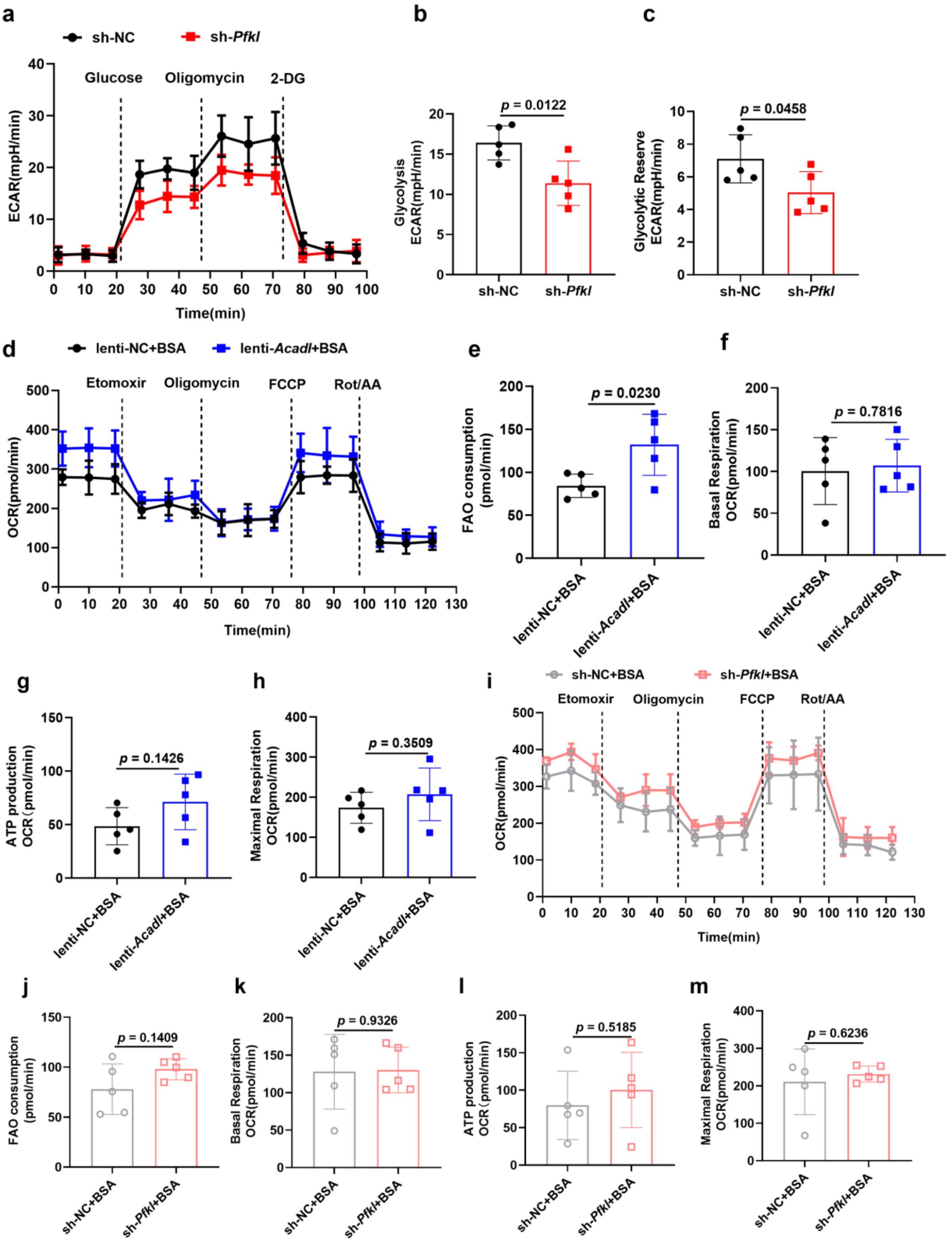

Supplementary Fig. 7. Knocking down *Pfkl* and overexpression of *Acadl* inhibited glycolysis and promoted FAO in cultured NMCMs, respectively. NMCMs stably transfected with control lentivirus (sh-NC) or knocking down PFKL lentivirus (sh-*Pfkl*) were supplied with 50 mM glucose, 1 μM oligomycin and 10 mM 2-DG at the indicated times. **a** ECAR was examined using Seahorse XF24 analyzer **b** Relative glycolysis levels and **c** glycolytic reserve capacity. **d** OCR over time using

BSA as the substrate in the control and overexpression of *Acadl* treated cultured cardiomyocytes, **e** FAO consumption, **f** Basal respiration, **g** ATP production , **h** maximal respiration (n = 5 biologically independent experiments). Assessment of OCR in cardiomyocytes treated with knockdown *Pfkl* and controls. **i** NMCMs were exposed sequentially to Etomoxir, Oligomycin, FCCP and Rot/AA, and OCR was measured over time using a Seahorse XF24 analyzer, cardiomyocytes FAO capacity test was performed according to manufacturer's protocol (n = 5 biologically independent experiments). There was no significant difference in (**j**) FAO consumption. (**k**) Basal respiration. (**l**) ATP production. (**m**) Maximal respiration between NMCMs treated with sh-*Pfkl* and sh-NC (n = 5 biologically independent experiments). Two-tailed unpaired Student's t-test in **b**, **c**, **e**-**h**, **j**-**m**. Data are depicted as the mean values ± SEM. sh, short hairpin; lenti, lentivirus overexpression; ECAR, extracellular acidification rate; OCR, oxygen consumption rate. Source data are provided as a Source Data file.

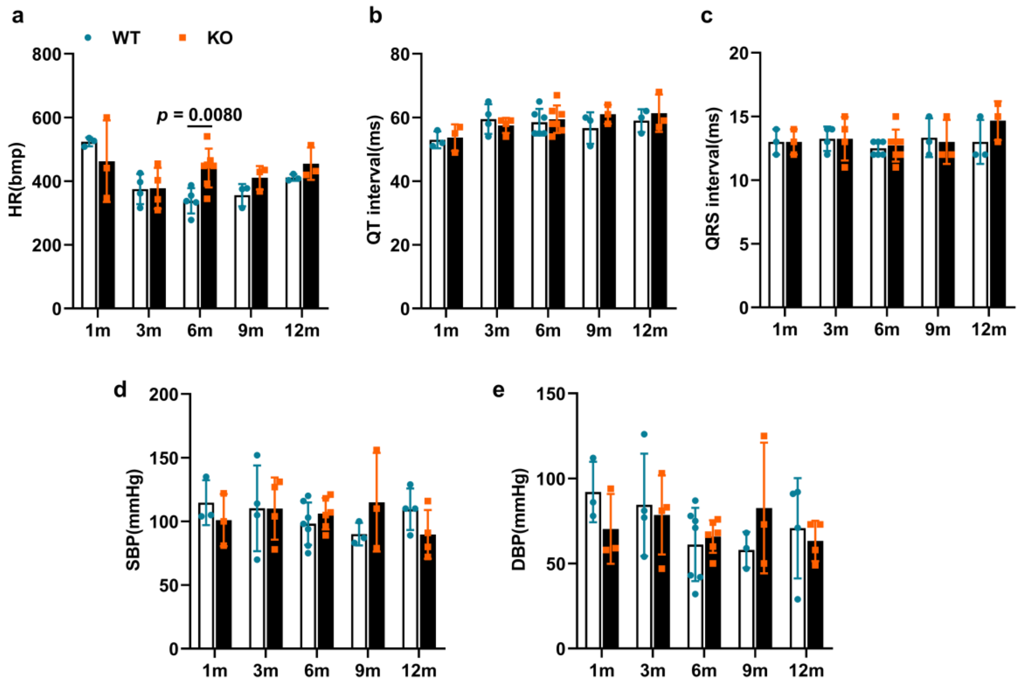

Supplementary Fig. 8. Cardiac-specific knockout of *Klf7* had no significant effect on ECG or blood pressure. **a** - **c** There was no difference heart rate, QT interval or QRS interval in ECG recordings from KO and WT mice (1m, n = 3 mice/group; 3m, n = 4 mice/group; 6m, WT, n = 6 mice/group, KO, n = 7 mice/group; 9m, n = 3 mice/group; 12m, n = 3 mice/group). **d**, **e** There was no difference in blood pressure at diastole or systole between the KO and WT mice (1m, n = 3 mice/group; 3m, n = 4 mice/group; 6m, n = 7 mice/group; 9m, n = 3 mice/group; 12m, WT, n = 3 mice/group, KO, n = 4 mice/group). Two-way ANOVA with Tukey's multiple comparison test in **a-e**. Data are depicted as the mean values ± SEM. HR, heart rate; SBP, systolic blood pressure; DBP, diasstolic blood pressure. Source data are provided as a Source Data file.

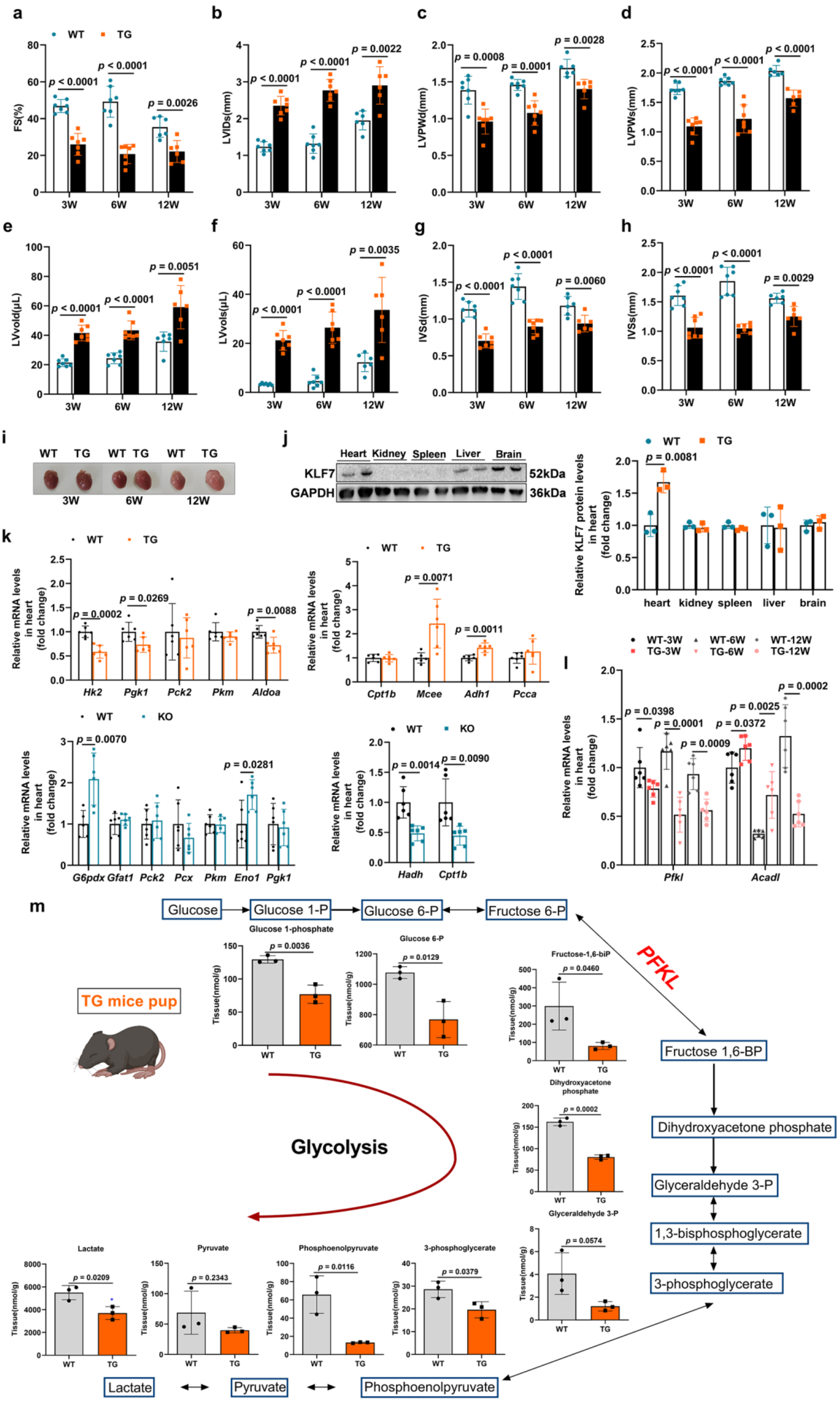

Supplementary Fig. 9. Cardiac-specific *Klf7* overexpression impaired cardiac

function and metabolism. Long-axis transthoracic M-mode echocardiographic traces from WT and TG mice at 3, 6, and 12 weeks of age were shown. When the echocardiographic parameters of KO mice were compared with those of WT mice, **a** FS was decreased (n = 7 mice/group), **b** end-systolic left ventricle inner diameter was increased (n = 7 mice/group), **c**, **d** end-diastolic and systolic posterior wall thickness was decreased (n = 7 mice/group), **e**, **f** end-diastolic and systolic LV volume was increased (n = 7 mice/group), and **g**, **h** end-diastolic and systolic IVS thickness was decreased (n = 7 mice/group). **i** Gross images of TG and WT mouse hearts at different periods. **j** Immunoblotting and quantification of cardiac-specific KLF7 protein expression in TG mouse tissues (n = 6 biologically independent experiments). **k** The expression of enzymes involved in disordered cardiac glucose and lipid metabolism in TG and WT mouse hearts identified by qRT-PCR (*Pgk1*, WT, n = 6 biologically independent samples, TG, n = 5 biologically independent samples; *G6pdx*, WT, n = 5 biologically independent samples, TG, n = 6 biologically independent samples; other genes, n = 6 biologically independent samples). **l** The mRNA levels of the *Klf7* target gene *Pfkl* were downregulated, while those of *Acadl* were upregulated in the myocardial tissues of TG mice compared to WT mice at different ages (n = 6 biologically independent experiments). **m** Pathway analysis of data obtained from mass spectrometry-based metabolomics showed that overexpression of *Klf7* significantly decreased glycolytic intermediates (n = 3 biologically independent samples). Two-tailed unpaired Student's t-test in **j**, **k** and **m**. One-way ANOVA with Tukey's multiple comparison test in **l**. Two-way ANOVA with Tukey's multiple comparison test in **a**-**h**. Data are depicted as the mean values ± SEM. KO, knockout *Klf7* mice; TG, overexpression *Klf7* mice. Source data are provided as a Source Data file.

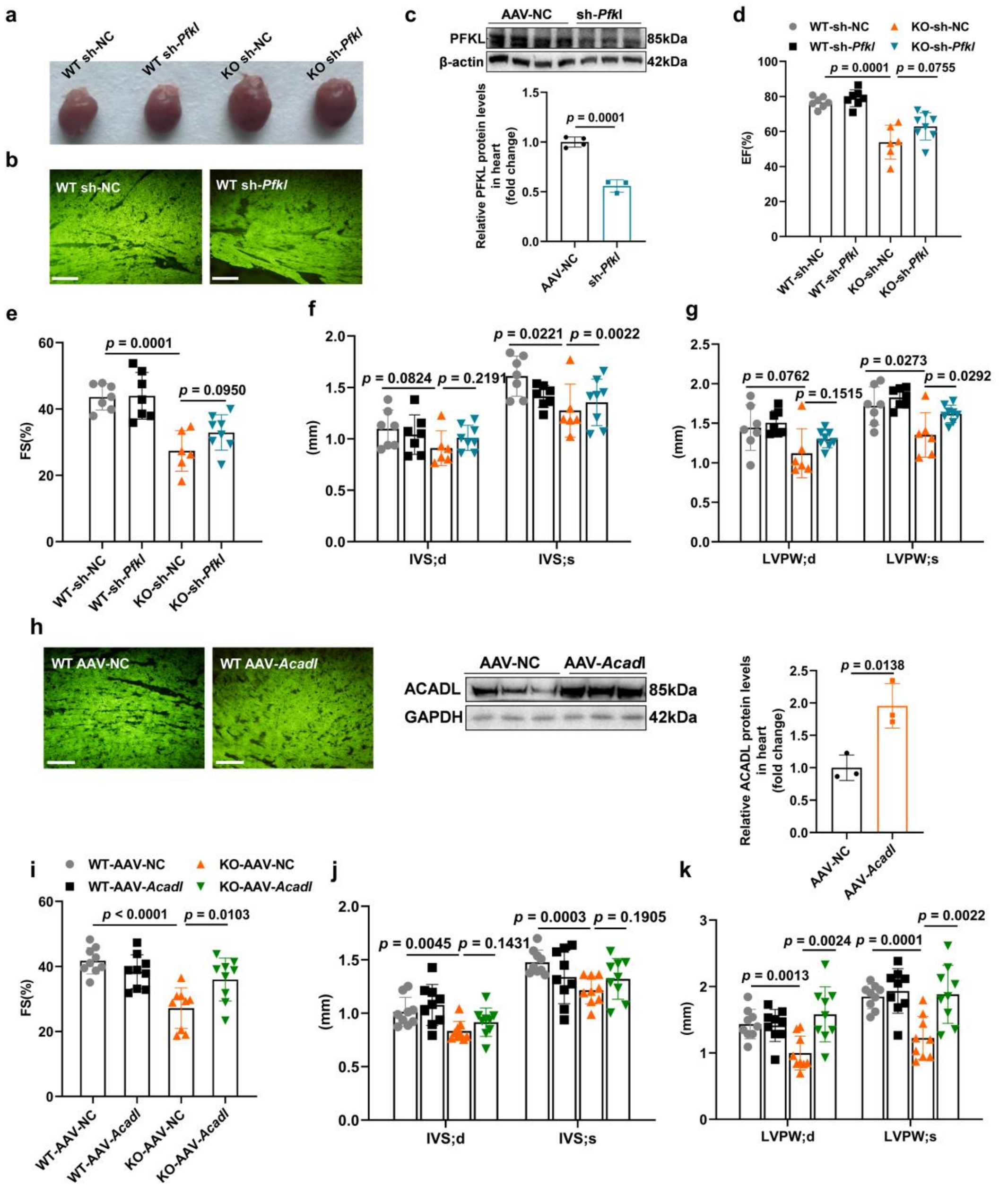

Supplementary Fig. 10. Cardiac-specific knockdown of *Pfkl* or overexpression of *Acadl* partially improved cardiac dysfunction in KO mice. **a** Representative gross image of the hearts of 6-month-old mice treated with sh-NC or sh-*Pfkl* for 4 weeks. sh-*Pfkl* reduced the heart volume in the *Klf7*^{KO} mice. **b** Fluorescence microscopy was used to detect the infection efficiency upon mouse tail-vein injection of AAV into myocardial tissue (n = 3 mice/group); scale bar: 500 μm. **c** The sh-*Pfkl*-mediated

myocardial tissue knockdown efficiency for PFKL was 60% (sh-NC, n = 4 biologically independent experiments, sh-*Pfkl*, n = 3 biologically independent experiments). **d-g** Echocardiographic parameters of the groups: the ejection fraction (EF), fraction shortening (FS), end-diastolic and systolic interventricular septum (IVSd/s), and end-diastolic and systolic posterior wall thickness (LVPWd/s) (n = 7 mice/group). **h** The infection efficiency upon mouse tail-vein injection of AAV for ACADL expression in myocardial tissue (n = 3 biologically independent experiments), scale bar: 500 μm. **i-k** Echocardiographic parameters of the groups: the LVvold/s, FS, IVSd/s, and LVPWd/s (n = 8 mice/group). Two-tailed unpaired Student's t-test in **c** and **h**. One-way ANOVA with Tukey's multiple comparison test in **d-g**, and **i-k**. Data are depicted as the mean values ± SEM. sh, knockdown with adeno-associated virus; AAV, adeno-associated virus; AAV-*Acadl*, overexpression *Acadl* with AAV. Source data are provided as a Source Data file.

Supplementary Table 1: Sequences of primer used for qPCR analysis

**gene name**	**primer sequences**
Hk2-for	TGATCGCCTGCTTATTCACGG
Hk2-rev	AACCGCCTAGAAATCTCCAGA
Gpi1-for	TCAAGCTGCGCGAACTTTTTG
Gpi1-rev	GGTTCTTGGAGTAGTCCACCAG
Aldoa-for	CGTGTGAATCCCTGCATTGG
Aldoa-rev	CAGCCCCTGGGTAGTTGTC
Tpi1-for	CCAGGAAGTTCTTCGTTGGGG
Tpi1-rev	CAAAGTCGATGTAAGCGGTGG
Gapdh-for	AGGTCGGTGTGAACGGATTTG
Gapdh-rev	TGTAGACCATGTAGTTGAGGTCA
Pgk2-for	TTCTGCTAAGTTGACTCTGGACA
Pgk2-rev	AGCCTTGATTCTCTGGTTGTTTG
Pgk1-for	ATGTCGCTTTCCAACAAGCTG
Pgk1-rev	GCTCCATTGTCCAAGCAGAAT
Pgam2-for	TGGAACCAAGAGAACCGTTTC
Pgam2-rev	TGGCATCTTTGATAGCGGTGG
Eno1-for	TGCGTCCACTGGCATCTAC
Eno1-rev	CAGAGCAGGCGCAATAGTTTTA
Pkm-for	GCCGCCTGGACATTGACTC
Pkm-rev	CCATGAGAGAAATTCAGCCGAG
*Pc*x-for	CTGAAGTTCCAAACAGTTCGAGG
Pcx-rev	CGCACGAAACACTCGGATG
Pck2-for	GTGGCCGTGCAATCCAGAA
Pck2-rev	GATGCCCAAAATCAGCATGTG
Pck1-for	CTGCATAACGGTCTGGACTTC
Pck1-rev	CAGCAACTGCCCGTACTCC
Cpt1b-for	GCACACCAGGCAGTAGCTTT
Cpt1b-rev	CAGGAGTTGATTCCAGACAGGTA
Acadl-for	TCTTTTCCTCGGAGCATGACA
Acadl-rev	GACCTCTCTACTCACTTCTCCAG
Gfat1-for	GAAGCCAACGCCTGCAAAATC
Gfat1-rev	CCAACGGGTATGAGCTATTCC
Pparα-for	AGAGCCCCATCTGTCCTCTC
Pparα-rev	ACTGGTAGTCTGCAAAACCAAA
G6pdx-for	CACAGTGGACGACATCCGAAA
G6pdx-rev	AGCTACATAGGAATTACGGGCAA
Hadh-for	TCAAGCATGTGACCGTCATCG
Hadh-rev	TGGATTTTGCCAGGATGTCTTC
Ldha-for	TGTCTCCAGCAAAGACTACTGT

Ldha-rev	GACTGTACTTGACAATGTTGGGA
Taldo1-for	GTGGGGCGCATCCTTGATT
Taldo1-rev	TGGTCTTGTAGCCGAACTTCT
Tkt-for	ATGGAAGGTTACCATAAGCCAGA
Tkt-rev	TGCAGCATGATGTGGGGTG
Decr1-for	GATCCGGGTCCTCAGAGGTTT
Decr1-rev	ATCAGGTGGTAGCATAGGCTT
Pcca-for	TTTGGCTAAAGCCGTGAAGTAT
Pcca-rev	GGATGTTCGACCTGTAGTCTTGT
Mcce-for	ATGAGGCGTGTAGTGAAGGC
Mcce-rev	TTTTCCAAATCTGGCACTGCTA
Adh1-for	GGAAAGCAACTTTTGTAGCCG
Adh1-rev	TACCACGGTGTACTGGGAGAA
Aldh2-for	CTGTTGTACCGATTGGCGGAT
Aldh2-rev	CGTAATAGCGGAGACATTTCAGG
Hmgcs2-for	AGAGAGCGATGCAGGAAACTT
Hmgcs2-rev	AAGGATGCCCACATCTTTTGG
Bdh1-for	GGTGGAACCTGGCAACTTCAT
Bdh1-rev	GGTCATCCCACATCTTCTTGG
Anp-for	GTGCGGTGTCCAACACAGAT
Anp-rev	TCCAATCCTGTCAATCCTACCC
Pfkl-for	GGAGGCGAGAACATCAAGCC
Pfkl-rev	GCACTGCCAATAATGGTGCC
Klf7-for	AGTGGACATTTTGCTCTCTCG
Klf7-rev	GTTAATGAGGTCACTGCGTTGA
Bnp-for	AGTCCTTCGGTCTCAAGGCA
Bnp-rev	CCGATCCGGTCTATCTTGTGC
Myh7-for	ACTGTCAACACTAAGAGGGTCA
Myh7-rev	TTGGATGATTTGATCTTCCAGGG
Ryr2-for	ACGGCGACCATCCACAAAG
Ryr2-rev	AAAGTCTGTTGCCAAATCCTTCT
Serca2a-for	TGGAACAACCCGGTAAAGAGT
Serca2a-rev	CACCAGGGGCATAATGAGCAG
Pln-for	AAAGTGCAATACCTCACTCGC
Pln-rev	GGCATTTCAATAGTGGAGGCTC
Tnnt2-for	CAGAGGAGGCCAACGTAGAAG
Tnnt2-rev	CTCCATCGGGGATCTTGGGT
Cacna1c-for	ATGAAAACACGAGGATGTACGTT
Cacna1c-rev	ACTGACGGTAGAGATGGTTGC
Caspase3-for	ATGGAGAACAACAAAACCTCAGT
Caspase3-rev	TTGCTCCCATGTATGGTCTTTAC
Tgfβ-for	CTTCAATACGTCAGACATTCGGG

Tgfβ-rev	GTAACGCCAGGAATTGTTGCTA
Col I-for	TAAGGGTCCCCAATGGTGAGA
Col I-rev	GGGTCCCTCGACTCCTACAT
Col III-for	CCTGGCTCAAATGGCTCAC
Col III-rev	GACCTCGTGTTCCGGGTAT
Nlrp3-for	ATTACCCGCCCGAGAAAGG
Nlrp3-rev	TCGCAGCAAAGATCCACACAG
