# Supplementary material for: The KLF7/PFKL/ACADL axis modulates cardiac metabolic remodelling during cardiac hypertrophy in male mice
Source: Nat Commun. 2023 Feb 21;14:959. doi: 10.1038/s41467-023-36712-9 (PMC9944323; doi:10.1038/s41467-023-36712-9)
Supplement: Supplementary file 3 — Reporting Summary [file 41467_2023_36712_MOESM3_ESM.pdf]

nature portfolio

Corresponding author(s): Weiming Tian

Last updated by author(s): Feb 4, 2023

Reporting Summary

Nature Portfolio wishes to improve the reproducibility of the work that we publish. This form provides structure for consistency and transparency in reporting. For further information on Nature Portfolio policies, see our Editorial Policies and the Editorial Policy Checklist.

Statistics

For all statistical analyses, confirm that the following items are present in the figure legend, table legend, main text, or Methods section.

n/a	Confirmed	
☐	☒	The exact sample size (*n*) for each experimental group/condition, given as a discrete number and unit of measurement
☐	☒	A statement on whether measurements were taken from distinct samples or whether the same sample was measured repeatedly
☐	☒	The statistical test(s) used AND whether they are one- or two-sided<br>*Only common tests should be described solely by name; describe more complex techniques in the Methods section.*
☐	☒	A description of all covariates tested
☐	☒	A description of any assumptions or corrections, such as tests of normality and adjustment for multiple comparisons
☐	☒	A full description of the statistical parameters including central tendency (e.g. means) or other basic estimates (e.g. regression coefficient) AND variation (e.g. standard deviation) or associated estimates of uncertainty (e.g. confidence intervals)
☐	☒	For null hypothesis testing, the test statistic (e.g. *F*, *t*, *r*) with confidence intervals, effect sizes, degrees of freedom and *P* value noted<br>*Give P values as exact values whenever suitable.*
☒	☐	For Bayesian analysis, information on the choice of priors and Markov chain Monte Carlo settings
☒	☐	For hierarchical and complex designs, identification of the appropriate level for tests and full reporting of outcomes
☒	☐	Estimates of effect sizes (e.g. Cohen's *d*, Pearson's *r*), indicating how they were calculated

Our web collection on statistics for biologists contains articles on many of the points above.

Software and code

Policy information about availability of computer code

Data collection	- ZEN (blue edition)<br>-Seahorse Wave Controller (version 2.6)<br>-Odyssey (version 3)
Data analysis	-Vevo LAB (version 3.2.0)<br>-GraphPad Prism (version 8.0)<br>-Image J (version 2)<br>-SOAPnuke (version 1.5.2)<br>-HISAT2 (version 2.0.4)<br>-StringTie (version 1.0.4)<br>-rMATS (version 3.0.9)<br>-Bowtie (version 2.2.5)<br>-RSEM (version 1.2.12)<br>-FlowJo (version 7.6.5)

For manuscripts utilizing custom algorithms or software that are central to the research but not yet described in published literature, software must be made available to editors and reviewers. We strongly encourage code deposition in a community repository (e.g. GitHub). See the Nature Portfolio guidelines for submitting code & software for further information.

Data

Policy information about availability of data

All manuscripts must include a data availability statement. This statement should provide the following information, where applicable:

- Accession codes, unique identifiers, or web links for publicly available datasets
- A description of any restrictions on data availability
- For clinical datasets or third party data, please ensure that the statement adheres to our policy

The RNA-seq data generated in this study have been deposited in the Zenodo database under accession code zenodo.5525482 [https://www.zenodo.org/record/5525482#.Y9XuasiExxs]. The targeted metabolome sequencing data generated in this study have been deposited in the Zenodo database under accession code zenodo.5525525 [https://www.zenodo.org/record/5525525#.Y9XtbciExxs]. The ChIP-seq data generated in this study have been deposited in the Zenodo database under accession code zenodo.5243430 [https://www.zenodo.org/record/5243430#.Y9d0vsiExxs]. The remaining data are available within the article, Supplementary information or Source Data file. Source Data are provided with this paper.

Human research participants

Policy information about studies involving human research participants and Sex and Gender in Research.

Reporting on sex and gender	N/A
Population characteristics	N/A
Recruitment	N/A
Ethics oversight	N/A

Note that full information on the approval of the study protocol must also be provided in the manuscript.

Field-specific reporting

Please select the one below that is the best fit for your research. If you are not sure, read the appropriate sections before making your selection.

☒ Life sciences ☐ Behavioural & social sciences ☐ Ecological, evolutionary & environmental sciences

For a reference copy of the document with all sections, see nature.com/documents/nr-reporting-summary-flat.pdf

Life sciences study design

All studies must disclose on these points even when the disclosure is negative.

Sample size	The sample size for animal studies was determined based on the numerous published study in the cardiac field (PMID: 6443464, 4886555, etc.) and our previous experience (PMID: 9547400). For cellular studies, the sample size was chosen according to preliminary experiments. At least three biologically independent replicates per group were collected to generate statistical testing.
Data exclusions	No data exclusion
Replication	All experiments were replicated as indicated in the respective figure legends.
Randomization	Mice were randomly assigned to each group and samples used in vitro experiments were randomly assigned to each group
Blinding	The investigators were blinded for mouse genotype and treatment during surgeries, echocardiography, organ weight determination and all histological and immunofluorescence quantifications. For other experiments, researchers were not blinded to group allocation during data collection due to the necessity of knowing the treatment to be administered or the samples to be collected.

Reporting for specific materials, systems and methods

We require information from authors about some types of materials, experimental systems and methods used in many studies. Here, indicate whether each material, system or method listed is relevant to your study. If you are not sure if a list item applies to your research, read the appropriate section before selecting a response.

Materials & experimental systems

n/a	Involved in the study
[ ]	[x] Antibodies
[ ]	[x] Eukaryotic cell lines
[x]	[ ] Palaeontology and archaeology
[ ]	[x] Animals and other organisms
[x]	[ ] Clinical data
[x]	[ ] Dual use research of concern

Methods

n/a	Involved in the study
[ ]	[x] ChIP-seq
[x]	[ ] Flow cytometry
[x]	[ ] MRI-based neuroimaging

Antibodies

Antibodies used

Primary antibodies:
1. Anti-ANP Rabbit pAb (1:1000; Abcam, catalog no. ab180649);
2. Anti-BNP Rabbit pAb (1:500, ABclonal, catalog no.A2179);
3. Anti-ACADL Rabbit pAb (1:1000, Abcam, catalog no. ab129711);
4. Anti-PFKL Rabbit pAb (1:1000, GeneTex, catalog no. GTX105697);
5. Anti-KLF7 mouse mAb (1:1000, Abnova, catalog no. H00008609-M01, clone no. 3E8-B8);
6. Anti-α-Tubulin mouse mAb (1:2000, ABclonal, catalog no. AC012, clone no. AMC0479);
7. Anti-β-actin Rabbit pAb (1:1000, ABclonal, catalog no. AC006);
8. Anti-GAPDH Rabbit pAb (1:1000, ABclonal, catalog no. AC001);
9. Anti-cardiac troponin T mouse mAb (1:200, Abcam, catalog no. ab8295, clone no. 1C11);
10. Anti-IgG Rabbit mAb (1:1000, Abcam, catalog no. ab172730, clone no. EPR25A);
11. Anti-HA-tag Rabbit pAb (1:500, Abcam, catalog no. ab9110);
12. Anti-CD45 mouse mAb (1:1000, BD Pharmingen, catalog no. 553079, clone no. 30-F11);
13. Anti-CD31 mouse mAb (1:1000, BD Pharmingen, catalog no. 558738, clone no. 390);
14. Anti-PDGFR-α mouse mAb (1:1000, BD Pharmingen, catalog no. 558774, clone no. APA5);
15. Anti-F4/80 mouse mAb (1:1000, BD Pharmingen, catalog no. 567893, clone no. T45-2342);
16. Anti-alpha smooth muscle Actin rabbit mAb (1:200, Abcam, catalog no. ab124964, clone no. EPR5368);
17. Anti-CD31 rabbit mAb (1:200, Abcam, catalog no. ab222783, clone no. EPR17260-263);
18. Anti-CD68rabbit mAb (1:200, Abcam, catalog no. ab53444, clone no. FA-11);
19. Anti-Vimentin rabbit mAb (1:200, Abcam, catalog no. ab92547, clone no. EPR3776).

Validation

All primary antibodies with satisfactory performance in this study were validated by the supplier in detecting target protein expression in mouse tissues, mouse cells and tissue section staining, and additional information including citations can be found on manufacture websites.

1. Primary antibodies provided by Abcam:
Anti-ANP Rabbit pAb and Anti-ACADL Rabbit pAb are validated by the supplier in detecting ANP and ACADL by Western blot. Anti-IgG Rabbit mAb and Anti-HA-tag Rabbit pAb are validated by the supplier in detecting HA-tag and IgG by IP. Anti-cardiac Troponin T mouse mAb, Anti-alpha smooth muscle Actin rabbit mAb, Anti-CD31 rabbit mAb, Anti-CD68rabbit mAb and Anti-Vimentin rabbit mAb are validated by the supplier on IF. Citations for these Abs can be found on the manufacturer's website by following antibody catalogue information. The performance of these Abs is presented in Figure 1g, 1h, 1m, 2a, 2b, 2f, 4f, 6f, 2c, 1l, 1p, 3b, 5c; Supplementary Figure 3g, 3d, 3h, 10h,5l.
2. Primary antibodies provided by ABclonal:
Anti-α-Tubulin mouse mAb, Anti-β-actin Rabbit pAb, Anti-BNP Rabbit pAb and Anti-GAPDH Rabbit pAb are validated by the supplier in detecting α-Tubulin, β-actin, BNP and GAPDH by Western blot. Citations for these Abs can be found on the manufacturer's website by following antibody catalogue information. The performance of these Abs is presented in Figure 1h, 1n, 1i, 1j, 1m, 1o, 2a, 2b, 2f, 3b, 4f, 6f, 1k, 1l, 1p, 5b; Supplementary Figure 3h, 3c, 3d, 3f, 3g, 9i, 10c, 10h.
3. Primary antibodies provided by Genetex:
Anti-PFKL Rabbit pAb is validated by the supplier in detecting PFKL by Western blot. Citations for PFKL antibody can be found on the manufacturer's website by following antibody catalogue information. The performance of the antibody is presented in Figure 1k, 1o, 2b, 3b, 5c; Supplementary Figure 3c, 3h, 10c.
4. Primary antibodies provided by Abnova:
Anti-KLF7 mouse mAb is validated by the supplier in detecting KLF7 by Western blot. Citations for KLF7 antibody can be found on the manufacturer's website by following antibody catalogue information. The performance of the antibody is presented in Figure 1j, 2a, 5b; Supplementary Figure 3f, 3h, 9j,
5. Primary antibodies provided by BD Pharmingen:
Anti-CD45 mouse mAb, Anti-CD31 mouse mAb, Anti-PDGFR-α mouse mAb and Anti-F4/80 mouse mAb are validated by the supplier on flow cytometry. Citations for CD45, CD31, PDGFR-α and F4/80 antibody can be found on the manufacturer's website by following antibody catalogue information. The performance of the antibody is presented in Supplementary Figure 5h.

Eukaryotic cell lines

Policy information about cell lines and Sex and Gender in Research

Cell line source(s)

Human cardiomyocyte AC16 cells (catalog no. BFN60808678) and human embryonic kidney HEK293T cells (catalog no. BFN60810479) were acquired from the Cell Bank of the Shanghai Academy of Chinese Sciencesl Bank

Authentication	Cell lines generally authenticated by morphology, specific structure, cell growth curve and division index, doubling time, inoculation rate, chromosome analysis, isozyme test, DNA fingerprinting, etc.
Mycoplasma contamination	We confirmed that all cell lines tested negative for mycoplasma contamination.
Commonly misidentified lines (See ICLAC register)	None

Animals and other research organisms

Policy information about studies involving animals; ARRIVE guidelines recommended for reporting animal research, and Sex and Gender in Research

Laboratory animals	Female and Male C56BL/6J mice (6 - 8 weeks old), Female KLF7KO mice (6 - 8 weeks old), αMHC-Cre mice (6 - 8 weeks old) and male TG mice (6 - 8 weeks old) were purchased from Cyagen Co., Ltd were applied for fertile. The KLF7KO and KLF7fl/fl mice (1 - 12 months) and TG and WT mice (3 - 12 weeks) were usually applied for the study of experiment. All the mice were kept in an environment that was free of pathogens (at 22 - 25℃, relative humidity of 45 – 60%, a 12 h light/dark cycle).
Wild animals	No wild animals were used in the study.
Reporting on sex	Several studies showed that risk factors and myocardial adaptations of cardiac hypertrophy and heart failure in men and women are different. Pre-menopausal women are better protected against cardiac hypertrophy and develop a more favorable physiological form of myocardial remodeling compared with men. Moreover, maladaptive left ventricular remodelling occurs more frequently in men and is associated with greater activation of profibrotic and inflammatory markers1-3. Collectively, we focused more attention on the pathological development of cardiac hypertrophy and heart failure in male mice.<br>Reference<br>1. Wu J , Dai F , Li C , et al. Gender Differences in Cardiac Hypertrophy. Journal of Cardiovascular Translational Research, 2019, 13(1).<br>2. Regitzzagrosek V . Sex and gender in myocardial hypertrophy. Wien Med Wochenschr, 2011, 161/5–6: 109–116<br>3. Georgios Kararigas, et al. Sex-dependent regulation of fibrosis and inflammation in human left ventricular remodelling under pressure overload. European Journal of Heart Failure, 2014, 16:1160–1167
Field-collected samples	No field collected samples were used in the study.
Ethics oversight	All animal experiments were approved and performed in accordance with guidelines set forth by the Harbin Institute of Technology Committee on Animal Resources (IACUC-2020035).

Note that full information on the approval of the study protocol must also be provided in the manuscript.

ChIP-seq

Data deposition

- [x] Confirm that both raw and final processed data have been deposited in a public database such as GEO.
- [x] Confirm that you have deposited or provided access to graph files (e.g. BED files) for the called peaks.

Data access links<br>*May remain private before publication.*	https://zenodo.org/record/5243430#.YgcahsiExxs
Files in database submission	S2 genes with peak, S2 marrow peaks ChIPseeker annotation, S2 peaks, S2 summits ChIPseeker annotation, S3 genes with peak, S3 narrow peaks ChIPseeker annotation, S3 peaks, S3 summits ChIPseeker annotation.
Genome browser session (e.g. UCSC)	UCSC

Methodology

Replicates	N2A cell lines n=2 independent experiments.
Sequencing depth	total number of reads: IP1 40557426, IP2 24526017,<br>uniquely mapped reads: IP1 33652576, IP2 20486990,<br>length of reads: IP1 242, IP2 227<br>they were paired,<br>the sequencing depth for each experiment is 6G.
Antibodies	Anti-IgG Rabbit mAb (1:1000, Abcam, catalog no. ab172730, clone no. EPR25A);<br>Anti-HA-tag Rabbit pAb (1:500, Abcam, catalog no. ab9110).
Peak calling parameters	Calculate the density of each chromosome (positive and negative chain) on the genome compared to Total mapped reads, as shown in the figure below. The specific mapping method is to use a sliding window (window size) of 5K, and calculate the internal comparison of the window to the base The median of reads at the base position and converted to log2; The regulatory mechanism of transcription factors and histones on genes is related to their binding positions, so analyzing the relative gene position distribution of

reads helps us predict the function of the protein. Divide each gene and the upstream and downstream 2K of the gene into 100 bins, count the number of reads falling in each bin, calculate the percentage of the number of reads in each bin to the total number of reads in these regions, as the read density of each bin; Use the computeMatrix module of the deepTools software to count the signals in the upstream and downstream 2kb region of the transcription start site (TSS). The specific process is as follows. Divide the entire area into bins according to the 50bp window size, and then calculate the average signal intensity in each bin; Use MACS2 software (Yong Zhang, Tao Liu et al., 2008) (threshold qvalue<=0.05) to complete peak calling analysis (peak calling), and count the number, width, distribution, etc. of peaks, and filter out peaks Related genes, etc.

Data quality

Raw data (raw reads) of fastq format were firstly processed using fastp software . In this step, clean data (clean reads) were obtained by removing reads containing adapter, reads containing ploy-N and low quality reads from raw data. At the same time, Q20, Q30 and GC content of the clean data were calculated. All the downstream analyses were based on the clean data with high quality.

Software

-fastp (version 0.19.11)
-RseQC (version 5.0.1)
-MACS2 (version 2.1.0)
-HOMER (version 3.13.3)
-ChIPseeker (version 4.0.0)
-DeepTools (version 2.0)
